# Does flying after embryo transfer impact implantation?

**DOI:** 10.5935/1518-0557.20250005

**Published:** 2025

**Authors:** João Antonio Dias Jr, Carlos Roberto Izzo, Georges Fassolas, Luiz Fernando Henrique, Denis Schapira Wajman

**Affiliations:** 1 Clínica Originare Medicina Reprodutiva, São Paulo, SP, Brazil

**Keywords:** embryo transfer, air travel, IVF outcomes, implantation, medical tourism

## Abstract

**Objective:**

To explore whether post-embryo transfer (ET) air travel affects in vitro fertilization (IVF) outcomes, addressing the growing concern in medical tourism where patients frequently travel for fertility treatments.

**Methods:**

This retrospective study was conducted at a private fertility clinic in São Paulo, Brazil, and included 2,135 embryo transfers performed between January 2019 and March 2022. Patients were divided into two groups: individuals who presumably traveled by airplane after ET (Group 2) and those who did not (Group 1). Data were analyzed for demographics, embryo transfer characteristics, and IVF outcomes. Relative risk was estimated using Poisson regression models, adjusting for age and treatment reasons. Significance was set at *p*<0.05.

**Results:**

17.94% of patients traveled by plane post-ET. No significant differences were found between the groups in terms of pregnancy (Group 1: 61.99%, Group 2: 62.14%, *p*=0.94), implantation (Group 1: 34.58%, Group 2: 38.65%, p=0.28), or miscarriage rates (Group 1: 28.51%, Group 2: 32.50%, *p*=0.32). A sub-analysis of single embryo transfers and top embryos also showed no statistical differences.

**Conclusions:**

Post-ET air travel did not negatively impact IVF treatment outcomes. Further prospective studies are recommended to confirm these findings.

## INTRODUCTION

Thousands of studies have tried to identify individual factors that could interfere with the success rate of In Vitro Fertilization (IVF) treatments, from preparation before stimulation to follow-up after the embryo transfer (ET). One of the most critical moments in fertility treatment is embryo transfer, which must be performed per specific management and follow-up guidelines. The guidelines of the ASRM (American Society of Reproductive Medicine) state that the literature for improving pregnancy rates supports the following interventions: abdominal ultrasound guidance for embryo transfer; removal of cervical mucus; use of soft embryo transfer catheters; placement of embryo transfer tip in the upper or middle (central) area of the uterine cavity, more than 1 cm from the fundus, for embryo expulsion; immediate ambulation once embryo transfer is completed (Practice Committee of the American Society for Reproductive Medicine, 2017).

In a globalized world, medical tourism has grown more substantial, and many patients travel to clinics in other countries to undergo fertility treatment (Cohen, 2014; Lunt & Carrera, 2010). Sometimes, patients must fly to the destination before and after treatment. This reality affects patients seeking different types of medical treatment (Bergmann, 2011; Salama *et al*., 2018). The question is whether air travel affects implantation after an embryo transfer.

Modern airplanes fly faster and at higher altitudes (Savona-Ventura & Mahmood, 2022). The cabin must be pressurized to allow passengers to travel in such conditions. Different mechanisms for pressurization were created, all involving changes to cabin pressure. Other risks associated with air travel include decreased humidity and radiation exposure (Savona-Ventura & Mahmood, 2022). Flying is safe for clinically pregnant patients (American College of Obstetricians and Gynecologists, 2002, Savona-Ventura & Mahmood, 2022).

Although recommendations for flying after an embryo transfer abound online, we have not found an indexed study on the subject, but a small one (Banerjee *et al*., 2017). The authors performed a retrospective analysis of 100 infertile females who underwent IVF from January 2016 to June 2016. They were divided into two groups - women who returned to their countries of origin three days after embryo transfer (48 individuals - Group 1) and women who stayed in the city where the treatment was performed (52 individuals - Group 2). The mean patient age, number of embryos transferred (1,2,3,4), grade (a or b), ease of transfer, and endometrial thickness were comparable in both groups. The pregnancy rate in group 1 was 60.4% (29/48) and 40.4% (21/52) in group 2, with a *p*-value of 0.045.

Although most recommendations indicate that flying is not harmful after an embryo transfer, the scarcity of scientific literature on the subject encouraged our group to examine our data for potential correlations.

The primary objective of our study is to explore whether post-embryo transfer air travel could impact the outcomes of IVF treatments. Our inquiry aims to provide a better understanding of this phenomenon and its potential implications for the IVF treatment process.

## MATERIALS AND METHODS

This is a single-center, retrospective study performed at a private fertility clinic (Originare Medicina Reprodutiva) in São Paulo, Brazil. The data collected from patient records were anonymized. The study included patients from January 2019 to March 2022, comprising 2,135 embryo transfers.

The study included patients aged 18 to 42 who underwent an embryo transfer (ET) and were divided into two groups according to whether they presumably flew after ET (Group 1) or not (Group 2).

Patients who received donor eggs or embryos were excluded. Positive HCG tests had levels equal to or greater than 5mIU/ml. Embryos rated as ≥3, AA, AB, and BA in the Gardner scale were graded as top embryos.

A low ovarian reserve was defined as an anti-Müllerian hormone (AMH) level below 1.2 ng/mL, based on the POSEIDON Group’s recommendation (Poseidon Group, 2016), in line with the Bologna Criteria from the ESHRE (Ferraretti *et al*., 2011). This threshold is similar to other studies referencing AMH cutoffs for successful egg donation (Oliveira *et al*., 2023).

We classified patients as likely to have flown if they lived more than 400 km from the clinic, implying they likely traveled by plane to return home. In such cases, the clinic did not restrict flying after embryo transfer.

Initially, the data were described through absolute frequencies and proportions (qualitative variables) and measures such as means, standard deviations, minimums, medians, and maximum values (quantitative variables). The Poisson regression model with repeated measures (Zou, 2004) and interactions between location and top embryo rating was used to estimate the crude and adjusted Relative Risk. Adjustments were made for age and reason for treatment. A log-link function was used to assess the relative risk (RR), and an identity-link function was used to estimate the average difference in percentage points. All analyses were performed using the SAS 9.4 software. A significance level of 5% was adopted.

## RESULTS

A total of 2,135 embryo transfers were included in the study. Of these, 1752 were in group 1 and 383 in group 2. An estimated 17.94% of patients presumably traveled by plane after embryo transfer and before the hCG blood test. We analyzed demographic variables, number of embryos transferred, quality of embryos transferred, reason for treatment, and treatment outcomes.

Tables 1, 2, and 3 provide demographic data and cycle characteristics. There was no statistical difference between the groups in these variables. On average, patients in group 1 had 1.71 embryos transferred compared to 1.59 in group 2. At least one top embryo was transferred in 22.60% of the procedures performed in group 1 *versus* 20.97% in group 2. Fresh transfers accounted for 15.53% and 18.54% of the procedures performed in groups 1 and 2, respectively.

**Table 1:** Demographics for couples seeking treatment.

Variables	Group 1 (Did not fly)			Group 2 (Flew)			*p* value^*^
	n	Mean (Std Dev)	Median (Min - Max)	n	Mean (Std Dev)	Median (Min - Max)	
Female Age	1752	36.35 (4.49)	36 (19 - 54)	383	36.03 (4.76)	36 (19 - 54)	0.16
Male Age	1701	39.24 (6.47)	38 (22 - 73)	367	38.78 (6.44)	38 (22 - 73)	0.17
Number of embryos transferred	1752	1.71 (0.63)	2 (1 - 4)	383	1.59 (0.59)	2 (1 - 4)	<0.01
% of top embryos transferred	1752	20.97 (36.67)	0 (0 - 100)	383	22.6 (38.11)	0 (0 - 100)	0.53

**Table 2:** Causes for infertility treatment.

	Group 1	Group 2	*p* value^**^
	(Did not fly)	(Flew)	
Low ovarian reserve	339 (19.35%)	75 (19.58%)	0.92
Uterine	86 (4.91%)	21 (5.48%)	0.64
Tubal disease	296 (16.89%)	68 (17.75%)	0.69
Endometriosis	347 (19.81%)	83 (21.67%)	0.41
Male	439 (25.06%)	103 (26.89%)	0.45
Adenomyosis	15 (0.86%)	2 (0.52%)	0.51
Anovulation	151 (8.62%)	35 (9.14%)	0.74
Unexplained	458 (26.14%)	109 (28.46%)	0.35
Same-Sex Couples	9 (0.51%)	2 (0.52%)	0.98
Genetic disorders	8 (0.46%)	1 (0.26%)	0.59

**Table 3:** Cycle characteristics.

Variable	Group 1	Group 2	*p* value^**^
	(Did not fly)	(Flew)	
IVF Fresh Thaw	272 (15.53%) 1480 (84.47%)	71 (18.54%) 312 (81.46%)	0.15
Number of embryos transferred 1 2 3 4	805 (45.95%) 875 (49.94%) 62 (3.54%) 10 (0.57%)	144 (37.6%) 210 (54.83%) 25 (6.53%) 4 (1.04%)	<0.01

The analysis of treatment outcomes is shown in Tables 4 and 5. Table 4 shows all cycles regardless of the number of embryos transferred and a comparison with similar-grade embryos, categorized as top embryos. Table 3 includes the same analysis for single-embryo transfers (SET) only. We separated SET and transfers with top embryos to mitigate confounding factors.

**Table 4:** Cycle results with any number of embryos transferred.

	Group 1 (Did not fly)	Group 2 (Flew)	Crude		Adjusted	
			Effect (95% CI)	*p* value	Relative risk (95% CI)	*p* value
Pregnancy rate [RR] General group At least one top embryo	1086 (61.99%) 360 (71.71%)	238 (62.14%) 74 (70.48%)	1 (0.92;1.08) 1.02 (0.89;1.16)	0.95 0.80	1 (0.91;1.0.9) 1.03 (0.89;1.20)	0.94 0.69
Implantation rate [Relative Mean] General group At least one top embryo	1006 (38.65) 348 (46.59%)	204 (34.58) 68 (43.04%)	1.12 (0.96;1.29) 1.08 (0.87;1.34)	0.12 0.47	1.08 (0.94;1.25) 1.06 (0.84;1.33)	0.28 0.64
Miscarriage rate [RR] General group At least one top embryo	189 (28.51%) 55 (26.83%)	52 (32.5%) 16 (30.77%)	0.88 (0.68;1.14) 0.87 (0.55;1.39)	0.32 0.56	0.88 (0.68;1.14) 0.87 (0.54;1.4)	0.32 0.56

**Table 5:** Cycle results for single embryo transfers.

	Group 1 (Did not fly)	Group 2 (Flew)	Crude		Adjusted	
			Effect (95% CI)	*p* value	Relative risk (95% CI)	*p* value
Pregnancy rate [RR] General group At least one top embryo	456 (56.65%) 140 (67.63%)	86 (59.72%) 24 (68.57%)	0.95 (0.82;1.1) 0.99 (0.77;1.26)	0.48 0.91	0.93 (0.8;1.08) 0.98 (0.76;1.26)	0.33 0.85
Implantation rate [RR]						
General group	341 (45.47%)	69 (87.5%)	0.89 (0.74;1.07)	0.20	0.87 (0.73;1.04)	0.13
At least one top embryo	98 (53.85%)	19 (59.38%)	0.91 (0.66;1.24)	0.54	0.88 (0.64;1.21)	0.42
Miscarriage rate [RR] General group At least one top embryo	56 (22.49%) 19 (26.03%)	13 (26.53%) 5 (35.71%)	0.85 (0.51;1.41) 0.73 (0.33;1.63)	0.53 0.44	0.87 (0.52;1.46) 0.87 (0.34;2.22)	0.59 0.77

Table 4 shows that group 2 had slightly better outcomes in pregnancy rate, 61.99% *vs*. 62.14% (*p*=0.94). A slight difference was seen in implantation rate, favoring group 2 (38.65 *vs*. 34.58% *p*=0,28). The miscarriage rate was slightly lower in group 1, 28.51% *vs*. 32.50% (*p*=0.32), although not significantly. When at least one top embryo was transferred, no statistical difference between groups was observed regarding pregnancy (71.71% *vs*. 70.48% *p*=0.69), Implantation (46.59% *vs*. 43.04% *p*=0.64), and miscarriage rates (26.83% *vs*. 30.77% *p*=0.56).

Table 5 shows no statistical difference between groups 1 and 2 in single embryo transfers. Regarding pregnancy rate, the difference was 56.65% *vs*. 59.72% (*p*=0,33) and 67.63% *vs*. 68.57% (*p*=0.85) for top embryos. For implantation, the difference was 45.47% *vs*. 51.11% (*p*=0.13) and 53.85 *vs*. 59.38% (*p*=0.42) for the general and top embryo analysis. Lastly, the relative risk for miscarriage rate was 0.87 (*p*=0.59) and 0.87 (*p*=0.77) when looking only at top embryos.

## DISCUSSION

The process of IVF is delicate and complex, involving various stages and interventions designed to optimize the chances of success. One of these key stages is the ET, which consists of the transfer of one or more embryos into the uterus of the woman undergoing treatment. The success of an ET depends on a range of factors, including the health of the embryos, the quality of the uterine lining, and the timing and conditions of the transfer itself. However, other factors could also pose challenges to the success of ET, such as flying on an airplane after the procedure, a topic that has not been adequately explored.

Medical tourism has grown to become a multibillion-dollar business (Cohen, 2014; Lunt & Carrera, 2010). Patients seek other places for treatment for many reasons, including cost, quality, and regulatory factors (the possibility of selecting gender and performing embryo analysis) (Bergmann, 2011; Salama *et al*., 2018). At least 20,000 to 25,000 couples are estimated to receive in-vitro fertilization (IVF) care annually in clinics located abroad (Peter, 2010), not to mention clinics within their country of origin but outside their home towns.

Given the increasing prevalence of air travel for medical treatment, it is essential to understand whether flying might pose a risk to patients undergoing IVF. While studies have examined the impact of air travel on fertility more broadly, limited research has been conducted specifically on the effects of flying after an ET (dos Santos Silva *et al*., 2009; Lauria *et al*., 2006; Mahoney, 2010).

Studies have shown that the embryo can move around after the transfer. The once common belief that patients must lie down after a transfer has proven pointless (Practice Committee of the American Society for Reproductive Medicine, 2017). Patients are generally concerned that external forces, such as those experienced during a flight, could disrupt the implantation process.

Newer airplane models are designed to minimize exposure to external factors such as radiation and atmospheric pressure changes, which may have previously been a concern. The longest commercial flights expose passengers to no more than 15% of the maximum annual radiation exposure (1mSv) (Barish, 2004). A 10-hour flight exposes passengers to 0.05mGy, while a chest x-ray exposes patients to twice as much radiation. A CT scan exposes patients to 8-30mGy of radiation (Savona-Ventura & Mahmood, 2022). When flying over 7,000m, passenger cabin pressure is generally maintained at the equivalent of an altitude of 1,524-2,438 m (Aerospace Medical Association, 2008). At such altitudes, passengers experience mild hypoxia, with resting oxygen saturation (SpO2) estimated at 90-95%, which is considered safe for healthy individuals (Silverman & Gendreau, 2009).

Another potential risk is exposure to whole-body scans used at airports during security checks. These scanners use low-energy, low-intensity ionizing radiation that does not penetrate the skin (Auvinen *et al*., 2012).

Several papers have analyzed the frequency of spontaneous miscarriages in flight attendants, with contradictory results (Aspholm *et al*., 1999; Cone *et al*., 1998; dos Santos Silva *et al*., 2009; Freeman *et al*., 2004; Grajewski *et al*., 2015; Lauria *et al*., 2006; Park *et al*., 2017). One study showed that flight attendants who flew more hours were more likely to have a miscarriage (Cone *et al*., 1998). Although fertility problems are more likely in this population, they appear to be linked to the job’s high physical job demands, lack of sleep, and disturbances in the circadian rhythm (Grajewski *et al*., 2015; Lauria *et al*., 2006; Mahoney, 2010; Mills & Kuohung, 2019).

Pregnant women are not exposed to greater risk because of flying when compared to women who did not choose air travel (Csorba *et al*., 2019; American College of Obstetricians and Gynecologists, 2002; Savona-Ventura & Mahmood, 2022). It is worth noting that those women flew significantly less than female flight attendants. The American College of Obstetricians and Gynecologists (ACOG) stated that occasional air travel is safe for pregnant women and has recommended measures to minimize potential risks (American College of Obstetricians and Gynecologists, 2002). In their review, the European Board and College of Obstetrics and Gynaecology (EBCOG) did not mention contra-indications to air travel for healthy pregnant women (Savona-Ventura & Mahmood, 2022).

While there is no definitive research on the impact of air travel after ET, the available evidence suggests that it is unlikely to worsen treatment outcomes (Banerjee *et al*., 2017). Our study also found no effect on ET from air travel.

As far as we know, this is the first study published in an indexed journal evaluating the potential impact of air travel after embryo transfer. The main strength of our research is the significant number of embryo transfer patients from a single center, which means that the technical protocols for endometrial preparation, embryo culture, embryo freezing and thawing, and the embryo transfer technique are similar and homogeneous.

However, this study has several limitations. Its retrospective nature is a primary weakness, as is the lack of control over whether patients took a flight as intended, the timing of airplane travel after the transfer, flight duration, the presence or absence of layovers, and potential flight difficulties such as turbulence. Furthermore, we could not quantify the cumulative birth rate due to the way data was extracted, which limits our ability to draw more definitive conclusions about overall reproductive success. Lastly, we could not compare the impact of different AMH levels across groups, which we understand could impact the results, especially in PCOS patients with high AMH levels (Vale-Fernandes *et al*., 2023).

It is important to note that further research is needed to confirm our findings. While evidence suggests that flying after an ET is safe, a prospective study is warranted to determine whether air travel impacts treatment outcomes.

We did not find negative impacts of air travel after embryo transfer. Allowing patients to return home after this procedure may reduce stress and treatment costs. Our data shows that patients should be encouraged to return home, even by airplane.

## CONCLUSION

Our study explored whether post-embryo transfer (ET) air travel could impact in vitro fertilization (IVF) outcomes. Given the growing prevalence of medical tourism and the need for patients to travel for fertility treatment, it is crucial to understand the potential risks associated with flying after an ET. Our retrospective analysis of 2,135 embryo transfers revealed no significant differences in pregnancy, implantation, or miscarriage rates between patients who traveled by airplane after ET and those who did not. While the study suggests that flying post-ET is generally safe, it is essential to acknowledge its limitations, including its retrospective nature and lack of control over flight-related variables. Therefore, further research, ideally prospective studies, must confirm these findings and provide definitive guidelines. Based on the available data, we conclude that air travel after ET does not negatively impact IVF treatment outcomes. This finding supports the notion that patients can safely return home by airplane after undergoing an embryo transfer, potentially reducing stress and treatment costs.
